# The System of Self-Consistent Models: The Case of Henry’s Law Constants

**DOI:** 10.3390/molecules28207231

**Published:** 2023-10-23

**Authors:** Andrey A. Toropov, Alla P. Toropova, Alessandra Roncaglioni, Emilio Benfenati, Danuta Leszczynska, Jerzy Leszczynski

**Affiliations:** 1 Laboratory of Environmental Chemistry and Toxicology, Department of Environmental Health Science, Istituto di Ricerche Farmacologiche Mario Negri IRCCS, Via Mario Negri 2, 20156 Milano, Italy; andrey.toropov@marionegri.it (A.A.T.); alessandra.roncaglioni@marionegri.it (A.R.); emilio.benfenati@marionegri.it (E.B.); 2 Interdisciplinary Nanotoxicity Center, Department of Civil and Environmental Engineering, Jackson State University, 1325 Lynch Street, Jackson, MS 39217-0510, USA; danuta@icnanotox.org; 3 Interdisciplinary Nanotoxicity Center, Department of Chemistry, Physics and Atmospheric Sciences, Jackson State University, 1400 J. R. Lynch Street, Jackson, MS 39217-0510, USA; jerzy@icnanotox.org

**Keywords:** Henry’s law constant, SMILES, QSPR, Monte Carlo method, system of self-consistent models, CORAL software

## Abstract

Data on Henry’s law constants make it possible to systematize geochemical conditions affecting atmosphere status and consequently triggering climate changes. The constants of Henry’s law are desired for assessing the processes related to atmospheric contaminations caused by pollutants. The most important are those that are capable of long-term movements over long distances. This ability is closely related to the values of Henry’s law constants. Chemical changes in gaseous mixtures affect the fate of atmospheric pollutants and ecology, climate, and human health. Since the number of organic compounds present in the atmosphere is extremely large, it is desirable to develop models suitable for predictions for the large pool of organic molecules that may be present in the atmosphere. Here, we report the development of such a model for Henry’s law constants predictions of 29,439 compounds using the CORAL software (2023). The statistical quality of the model is characterized by the value of the coefficient of determination for the training and validation sets of about 0.81 (on average).

## 1. Introduction

The constants of Henry’s law are desired for assessing the processes related to atmospheric pollutants, particularly those related to their transport in the atmosphere. Chemical changes in the gaseous phase affect the fate of atmospheric pollutants and ecology, climate, and human health.

Quantitative structure–property relationships (QSPRs) are one of the tools applied to describe physicochemical endpoints, which characterize the status of the earth’s atmosphere. Such endpoints could be, for example, the rate constants for the reaction of OH radicals, the rate constants of reactions of ozone with organic and inorganic compounds, vapor pressure, and Henry’s law constants [1].

The experimental measurements for Henry’s law constants are reported using different techniques, including headspace gas chromatography, modified headspace techniques, phase ratio variation, the differential headspace method, and dilution techniques [2]. However, accurate Henry’s law constants values are currently unavailable for many compounds. Instrumental problems make the experimental determination of Henry’s law constants values difficult and expensive. Consequently, applying theoretical methods to robustly predict this endpoint for diverse compound types is essential [2].

Global climate change, attributed to the increased levels of greenhouse gases produced by the use of fossil fuels, is recognized as the most important challenge of nowadays. The increasing concentration of greenhouse gases in the atmosphere contributes to global warming, which already contributes to the increasing extreme weather events. Mitigating climate change has proved very difficult due to complex distribution problems across resources for different countries. In the future, geochemical and geophysical impacts are expected to cause significant harm to ecosystems.

The use of regulatory impact assessment is increasingly becoming a standard procedure in OECD countries to prepare regulations. As noted above, climate impact assessment is one of the priority objects of regulatory activity around the world, aimed at controlling and observing climate change [1,2]. Since Henry’s law constants are a source of valuable information on the environmental effects of organic compounds, QSPRs can serve as a tool to simulate the above constants [3,4,5]. 

It is necessary to take into account that in addition to the knowledge or at least estimation of numerical values on Henry’s law constants, computer experiments aimed at obtaining such data as they develop and improve can be a source of additional information helping to formulate and test theoretical ideas about the nature of the influence of molecular architecture by the values of the mentioned constants. This, in turn, can provide the basis for finding technological solutions that are less dangerous in terms of impact on the environment, as well as on human health and farm animals and plants.

Cross-validation was used initially to estimate the prediction error of a mathematical modeling procedure. For a couple of decades, it was trusted that cross-validation estimates the prediction error unbiasedly. Nonetheless, numerous reports in the cheminformatics literature show that cross-validated figures of merit cannot be trusted. Instead of cross-validation, other variants of algorithms are possible that can provide the user with statistical measures of model reliability, or, more precisely, measures of reliability of the predictive potential of models. The essence of such algorithms is to use many options for distributing the available data into non-overlapping training and control samples. One can carry out such distributions in any way one likes, but most likely, the most “reliable” or at least the most impartial are random divisions into the above-mentioned sets. Another important condition, if unsuccessful, then at least with greater confidence in the model verification carried out this way, is the consideration and comparison of many such random distributions. The more such divisions into training and control are considered, the more reasons there are to read such assessments as reliable. Such verification is referred to in the literature as a system of self-consistent models [6]. Unlike previous approaches used to test/evaluate the predictive potential of models, the system of self-consistent models is also a method for constructing a model. Due to the significant contribution of random transformations of available data carried out when building such models, the results obtained can hardly be assessed as artificial. They are as natural as chance itself. However, this “naturalness” contains not only attractive but also unpleasant possibilities, which are a consequence of the slow increase in accuracy/reliability with an increase in the number of corresponding computer experiments. The Monte Carlo method is suitable for analyzing and comparing the mentioned random distributions in training and validation sets. Still, it also leads to the need to consider “extra” unsuccessful distributions. By taking these “excess experiments” into account, the corresponding comparisons are characterized by significant dispersion, for a fair assessment of which a large number of corresponding samples (models) is necessary.

The present study reports the results of an attempt to apply a system of self-consistent models [6,7] to assess the predictive potential of models of Henry’s law constants simulated by the CORAL software (http://www.insilico.eu/coral, accessed on 12 October 2023).

## 2. Results and Discussion

### 2.1. The System of Self-Consistent Models

The system of self-consistent models represents the process of testing the predictive potential of an applied approach. It involves building models with several random distributions of the available data into training and validation sets. Perhaps the process could be considered a variation of the two-deep cross-validation [8]. The process can be represented as the following:

(1) $$\begin{array}{ccc} \begin{array}{cc} M_{1}:V_{1}^{*}\to R_{1,1}^{2*} & M_{1}:V_{2}^{*}\to R_{1,2}^{2*}\\ M_{2}:V_{1}^{*}\to R_{2,1}^{2*} & M_{2}:V_{2}^{*}\to R_{2,2}^{2*} \end{array} & \cdots & \begin{array}{c} M_{1}:V_{n}^{*}\to R_{1,n}^{2*}\\ M_{2}:V_{n}^{*}\to R_{2,n}^{2*} \end{array}\\ \vdots & \ddots & \vdots \\ \begin{array}{cc} M_{n}:V_{1}^{*}\to R_{n,1}^{2*} & M_{n}:V_{2}^{*}\to R_{n,2}^{2*} \end{array} & \cdots & M_{n}:V_{n}^{*}\to R_{n,n}^{2*} \end{array}$$

**Figure 1:**
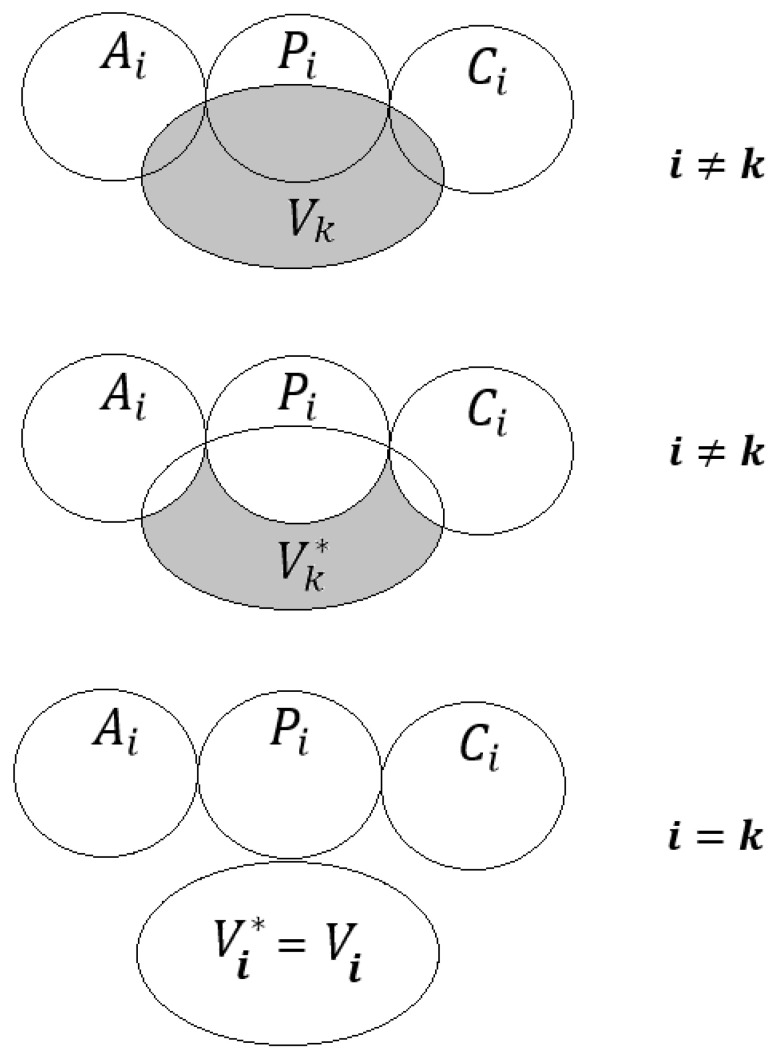
The principle of the selection of the list of compounds from the validation set of the *k*-th split to confirm the predictive potential of the *i*-th model. Symbol ‘*’ denotes the subset of compounds of the k-th validation set of k-th split without compounds that are included in the active training set, passive training set, or the calibration set of the i-th split.

M_i_ represents the *i*-th model calculated with Equation (3) with the correlation weights obtained by the Monte Carlo optimization, which gives a maximum of the target function calculated with Equation (5). V_k_ is the list of compounds distributed to the validation set in a *k*-th split of data. Figure 1 shows the principle of the selection of compounds for the *i*-th model validation with a *k*-th split validation set.

### 2.2. The Statistical Quality of Models

The model of pHLC observed in the case of split 1 is the following:

pHLC = 3.8465 (±0.0005) + 1.1055 (±0.0001) × DCW(3.15)(2)

**Figure 2:**
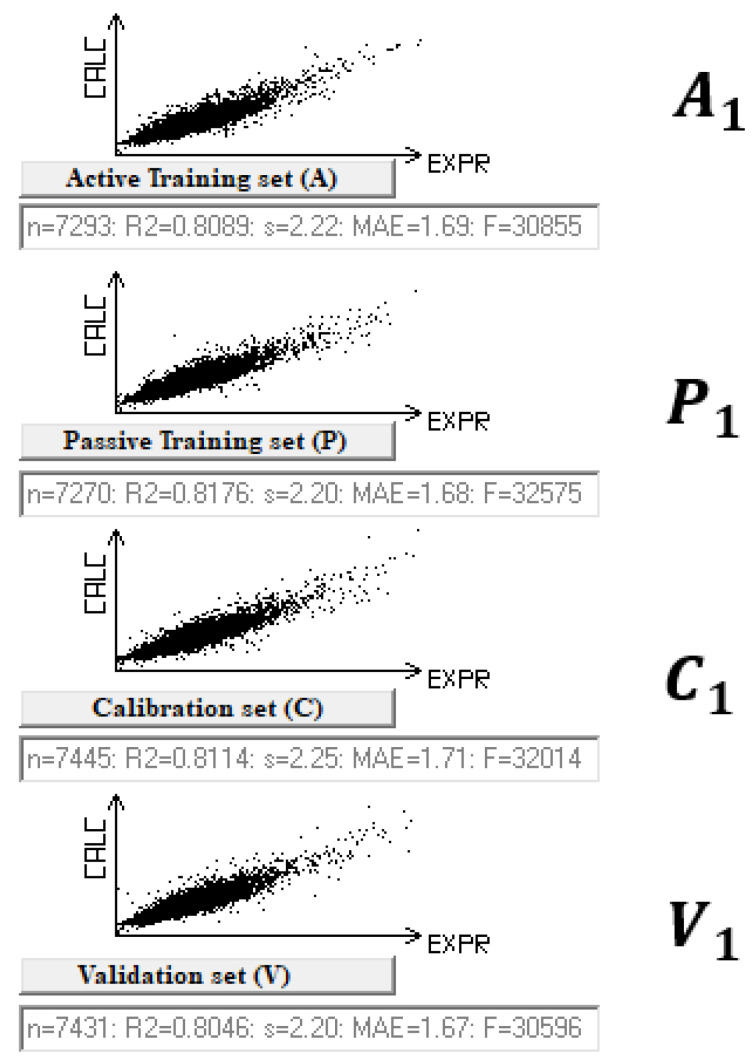
The graphical representation of the model for pHLC was observed in the case of split 1. *A*_1_, *P*_1_, *C*_1_, and *V*_1_ are the active training, passive training, calibration, and validation sets, respectively.

Figure 2 shows the results of the model graphically.

Table 1 contains the statistical quality of models observed for ten random splits, showing several statistical parameters. The values of these parameters are quite similar for the different splits, and this indicates that the methodology is robust and replicable. This fact is reasonably expected due to the large number of substances within each split; thus, we can assume that the distribution of the substances within each split is representative of the general behavior, and there are not many substances with deviating values. The large number of substances facilitates the task; still, the spread of values for Henry’s constant is quite large, over N orders of magnitude. Thus, despite the modeling difficulties, the developed model is a good one. Furthermore, within each split, the values related to the different sets (active training, passive training, calibration, and validation) are also quite constant and good, and this is another demonstration of the quality of the model, which is expected to provide the same kind of performance when used on new substances. For instance, the R^2^ values are good, around 0.8.

**Figure 3:**
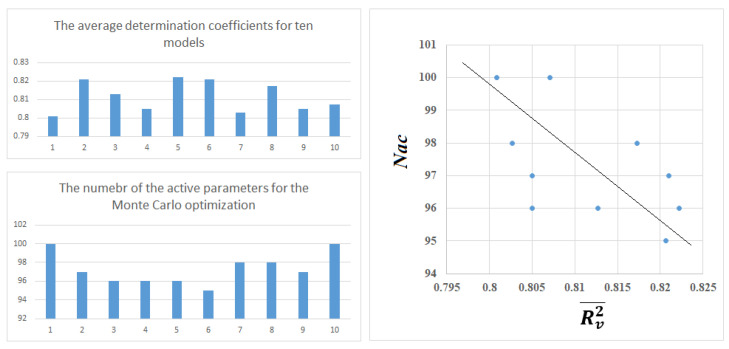
Anti-correlation between the number of active SMILES attributes involved in the Monte Carlo optimization and the average determination coefficient of a model’s overall splits.

Table 2, Table 3 and Table 4 represent the system of self-consistent models. Despite the similarity of the statistical quality of the models, one can see that there is a difference. Various distributions of available data in training (i.e., A, P, and C) and validation sets resulted in somehow different models, and some of them are better than others. Figure 3 shows that in the overall splits, there is an anti-correlation between the number of SMLES attributes involved in the Monte Carlo optimization and average values of determination coefficients for the validation sets of the models. Thus, a paradoxical situation is observed: a smaller number of optimized parameters is accompanied by an improvement in the predictive potential of the model. A possible explanation is that there is a preferred number of inputs to the model in order to be generally correct, and by adding more inputs, there is a loss of predictivity due to overfitting [12].

### 2.3. Why Are Models Needed?

The simplest and most traditional ideas for answering the question highlighted in the title are that the experiment is expensive and takes time to complete. However, this is just the tip of the iceberg. There are quite deep needs for building models in the aspect of epistemology. Since practice is the basis of the development of society, the development of various models is dictated primarily by the need for practical solutions to real problems, and here, problems arise that are much more unpleasant and complex than the definitions of the domain of applicability and unambiguity of algorithms. You should start by determining who the consumer of the model is. The complication of models is rarely accompanied by their actual improvement. As a result, the consumer of the model, despite being interested in using the model, often simply does not know how to do it. Under such circumstances, it becomes likely that the only user of the model remains the developer of this model. To avoid this simple, unpleasant situation, significant efforts are needed on the part of model developers. All models are wrong [13], and only some of them are useful. How do we recognize useful models? It seems that not the last condition for the usefulness of a model is its popularity. In other words, the model is useful if it is used. However, popularity does not mean usefulness. At least without the useful results of using the model, it cannot be considered useful. Many factors determine the usefulness of a model, but the first one is the clarity of the result or the ability to assess how reliable the outcome is quickly. The reliability of a result starts with its reproducibility. Often, reproducibility may not be absolute and is accompanied by some level of variance in the result, such as the statistical quality of the model expressed in terms of the coefficient of determination and root mean squared error. However, in reality, the model for the endpoint of interest is the value extracted from complex computer experiments obtained for the only correct distribution of the available data into the training and validation subsystems. What is bad about it? First of all, the possibility for such a model to turn out to be a beautiful accident does not coincide with the real difficulties and paradoxes of predicting the endpoint in question; of course, this situation is especially “dangerous” when few data are available. If there are a lot of data, the probability that some selected split into training and validation is successful becomes smaller the greater the amount of data available for model development. The result is a situation similar to the uncertainty principle: the more data, the more reliable the result, which, however, is most likely less accurate. In other words, when determining a model from a small number of available data, the coefficient of determination will be close to 1. Still, when determining the same model for a large number of data, the coefficient of determination will not be comparable to 1. Since all models are wrong, the researcher, whether a model developer or a model user, must be on guard; that is, to avoid a situation where fighting mice (high prediction accuracy) distracts from the “tiger” present (the impossibility of contradicting the uncertainty principle or the contradiction between the need to consider large amounts of experimental data, which guarantees an increase in uncertainty in the forecast). All this leads to the triumph of seditious thoughts, which is bad if there are few data and equally bad if there are too many data. This means there must be some kind of “correct” reliable middle ground where there are exactly as many data as needed for a useful model (the model that is still incorrect).

### 2.4. Model as a Hired Worker in a Workshop

Almost always, the model acts as a kind of assistant in solving various problems. Here, a certain analogy arises with the cooperation between a hired worker and the owner of a certain workshop or even production. If following this analogy, like a worker, the model must have certain capabilities and qualities. A model must be able to do something. In the context of the QSPR, the model must be able to, having received a standard task (input data), predict something corresponding (the expected value) to the input data that are offered to it. At the same time, a hired worker should not take on arbitrary tasks but only those for which they are an expert. Likewise, the model should abandon a problem that it cannot reliably solve. The model should at least abandon the attempt to solve the impossible task, but it is better if the model is able to explain why this problem cannot be solved using this model. This leads to the formulation of the quite important concept of QSPR simulation known as the “applicability domain”. Typically, the applicability domain is calculated based on distances in the multidimensional descriptor space or based on the similarity of molecular graphs. However, another possibility is for the user to determine the scope of the applicability of the model. It would be ideal to determine the applicability domain during the process of building the model; that is, taking into account the wishes of the potential user of the model. In other words, the model is self-sufficient, able to regulate and adequately evaluate its actions by the “proposed task”.

### 2.5. A Model Is Either Knowledge or Delusion

A model can be knowledge if certain conditions for its development and use are met. The model must be carefully tested. All models are wrong [13]. All models are random events [14]. For a model to be useful, it must work to create new knowledge. For a model to produce knowledge, its results must be reproducible according to elementary logic.

The approach considered here (the system of self-consistent models) is an attempt to create, or at least simulate, the ability of a model, independent of the user, to “check its actions”. The self-consistency means not only (or even not so much) the similarity of the statistical quality of the models but rather the verification of the predictive potential on compounds really “invisible” in the process of developing the model (i.e., compounds selected according to the scheme that is shown in Figure 1).

It may seem that the system of self-consistent models is an approach conceptually similar to cross-validation [15]. However, if within the framework of cross-validation, one model is used in which the effect of removing one or several (e.g., 5-fold [16]) participants (compounds) is studied, then in the case of systems of self-consistent models, the predictive potential of many models built based on different training sets is studied and compared. The proposed approach will undoubtedly provide a departure from the naive Q^2^ [8] and make it possible to evaluate the predictive potential in terms of the index of the ideality of correlation, which incorporates information about both the determination coefficient and the mean absolute error (MAE).

Similar to the world of material movements, in which all events visible and tangible to us in everyday life take place, such as traffic, sports, or exchange rates, there is a world of probabilistic actions, accidents, and even catastrophes that influence each other. However, these are not visible and not tangible to us. Perhaps quantitative structure–property/activity relationships (QSPRs/QSARs) allow you to look into this world of accidents and trends that affect each other. There is no mysticism here, but the phenomena occurring in such a space are not always described ideally and reliably. In other words, encountering situations that, in contrast to logic, are possible and unpleasant. For example, the quality of calculations (models) can be affected by the collection of substances that are available in the database or a list of priorities and criteria selected in the software used to build up QSPRs. However, in any case, it remains an indisputable axiom that models of random events are knowledge only when they are understandable and allow the possibility of verification by establishing and confirming their reproducibility.

The use of various descriptors to predict the physicochemical properties of substances has been criticized many times. This criticism covered many aspects of the QSPR use. The main point of criticism was the very idea of correlation. That is, correlation does not show causes. Another point of criticism is the impossibility of accurately determining the applicability domain. However, the practical use of QSPRs is increasing rather than decreasing. Two circumstances able to improve the quality of discussion between critics and supporters of QSPRs: (i) it cannot be expected that QSPRs are capable of replacing experiments; and (ii) a QSPR can become undeniably useful if QSPRs become the language of communication between the experimenter and the developer of a QSPR, not a mathematical tool itself.

Finally, last but not least, the proposed approach is implemented through one program available on the internet (http://www.insilico.eu/coral, accessed on 12 October 2023), and that has been used as a tool to develop QSPR/QSAR models many times [17,18,19,20,21,22,23,24].

## 3. Method

### 3.1. Data

Experimental Henry’s law constants (Atm m^3^/mole) were obtained from [1] as expressed via negative decimal logarithm (pHLC). They were examined as the endpoint for QSPR analysis. The large experimental data set (*n* = 29,439) was applied to distribute experimental data into four sets using ten different splits. The resulting data sets included the active training-A (≈25%), passive training-P (≈25%), calibration-C (≈25%), and validation-V (≈25%) sets (the average percentage of similarity for these splits was less than 30%). Each of the above sets has a defined task. The active training set is used to build the model: molecular features extracted from the SMILES of the active training set are involved in the process of Monte Carlo optimization aimed at providing correlation weights for the above features, which give a maximal target function calculated using descriptors (the sum of the correlation weights) and the endpoint on the active training set. The task of the passive training set is to check whether the model obtained for the active training set is satisfactory for the SMILES, which was not involved in the active training set. The calibration set should detect the start of the overtraining (overfitting). At the beginning of the optimization, the correlation coefficients between the experimental values of the endpoint and the descriptor contemporaneously increase for all sets. Still, the correlation coefficient for the calibration set reaches the maximum (this is the start of the overtraining), and further optimization leads to a decrease in the calibration set’s correlation coefficient. The optimization should be stopped when overtraining starts. After stopping the Monte Carlo optimization procedure, the validation set is used to assess the predictive potential of the obtained model.

### 3.2. Model

The model values of Henry’s law constants were calculated as

(3) $$\mathrm{pHLC}=C_{0}+C_{1}\times \mathrm{DCW}\left(3.15\right)$$

where C_0_ and C_1_ are regression coefficients; the optimal SMILES-based descriptor DCW applies two thresholds; the first threshold equal to 3 is used to define the active and inactive attributes of the simplified molecular input-line entry system (SMILES); and the second threshold, equal to 15, refers to the number of epochs in the Monte Carlo optimization process [25]. See Supplementary Materials Table S1–S4.

### 3.3. Optimal Descriptor

Active SMILES attributes have so-called correlation weights (CWs), which are necessary to calculate the descriptor DCW(3.15) by the formula:

(4) $$\mathrm{DCW}\left(3.15\right)=\sum \mathrm{CW}(S_{k})+\sum \mathrm{CW}\left({\mathrm{SS}}_{k}\right)$$

Table 5 contains an example of the calculation of the DCW(3.15).

### 3.4. The Monte Carlo Optimization

Equation (4) needs the numerical data on the above correlation weights. Monte Carlo optimization is a tool to calculate those correlation weights. The following target functions for the Monte Carlo optimization are used:

(5) $${\mathrm{TF}}_{\;}=r_{\mathrm{AT}}+r_{\mathrm{PT}}-\left|r_{\mathrm{AT}}-r_{\mathrm{PT}}\right|\times 0.1$$

The $r_{\mathrm{AT}}$ and $r_{\mathrm{PT}}$ are correlation coefficients between the observed and predicted endpoints for the active and passive training sets.

## 4. Conclusions

In this work, the application of the system of self-consistent models, which are generated by the CORAL software, was used to develop a predictive model based on a large set of substances with values of Henry’s law constants. The suggested version of the optimal descriptor and the Monte Carlo optimization provide satisfactory predictive potential for all ten random splits of the experimental data. Nevertheless, the model self-consistency system showed that though the predictive potential of the models does not differ too much, this difference is noticeable. In addition, reducing the number of optimized parameters improves the predictive potential of the model (Figure 3). This circumstance can be used to determine the quality of the partition used: the fewer parameters to be optimized, the more likely it is to improve the predictive potential of the model.

## Figures and Tables

**Table 1:** The statistical quality of models observed for ten random splits into the active training (A), passive training (P), calibration (C), and validation (V) sets.

*Split*	*Set*	n*	*R* ^2^	*CCC*	*Q* ^2^_*F*1_	*Q* ^2^_*F*2_	*Q* ^2^_*F*3_	<*R_m_*^2^>	*RMSE*	*MAE*	*F*	*Nac*
1	A	7293	0.8089	0.8943					2.22	1.69	30,855	
	P	7270	0.8176	0.8980					2.20	1.68	32,575	
	C	7445	0.8114	0.8957	0.8114	0.8114	0.8060	0.7341	2.25	1.71	32,014	
	V	7431	0.8046	-	-	-	-	-	2.20	1.67	-	100
2	A	7374	0.8288	0.9064					2.10	1.59	35,690	
	P	7156	0.8223	0.9019					2.14	1.61	33,112	
	C	7410	0.8226	0.9023	0.8226	0.8225	0.8173	0.7502	2.17	1.61	34,345	
	V	7499	0.8239	-	-	-	-	-	2.15	1.62	-	97
3	A	7258	0.8160	0.8987					2.14	1.62	32,179	
	P	7371	0.8187	0.9006					2.19	1.66	33,278	
	C	7473	0.8217	0.9015	0.8217	0.8217	0.8140	0.7425	2.18	1.65	34,436	
	V	7337	0.8157	-	-	-	-	-	2.19	1.64	-	96
4	A	7335	0.8174	0.8995					2.18	1.65	32,824	
	P	7364	0.8174	0.8987					2.18	1.65	32,955	
	C	7336	0.8293	0.9065	0.8293	0.8293	0.8243	0.7589	2.14	1.63	35,629	
	V	7404	0.8002	-	-	-	-	-	2.25	1.71	-	96
5	A	7431	0.8248	0.9040					2.13	1.61	34,978	
	P	7369	0.8187	0.9001					2.13	1.62	33,268	
	C	7313	0.8305	0.9078	0.8305	0.8305	0.8221	0.7617	2.13	1.60	35,828	
	V	7326	0.8239	-	-	-	-	-	2.15	1.63	-	95
6	A	7348	0.8130	0.8968					2.16	1.63	31,931	
	P	7413	0.8227	0.9028					2.16	1.65	34,391	
	C	7346	0.8149	0.8968	0.8148	0.8148	0.8065	0.7291	2.23	1.66	32,334	
	V	7332	0.8240	-	-	-	-	-	2.14	1.62	-	98
7	A	7264	0.8068	0.8931					2.23	1.70	30,321	
	P	7338	0.8068	0.8935					2.26	1.73	30,627	
	C	7453	0.8021	0.8911	0.8019	0.8019	0.8036	0.7230	2.26	1.73	30,191	
	V	7384	0.8030	-	-	-	-	-	2.26	1.74	-	98
8	A	7184	0.8232	0.9030					2.16	1.63	33,431	
	P	7413	0.8262	0.9039					2.14	1.61	35,239	
	C	7284	0.8335	0.9083	0.8334	0.8334	0.8293	0.7595	2.12	1.60	36,444	
	V	7558	0.8132	-	-	-	-	-	2.14	1.62	-	97
9	A	7470	0.8064	0.8928					2.27	1.73	31,109	
	P	7312	0.8002	0.8898					2.26	1.72	29,275	
	C	7389	0.7884	0.8823	0.7885	0.7884	0.7966	0.7043	2.31	1.75	27,523	
	V	7268	0.8067	-	-	-	-	-	2.27	1.74	-	100
10	A	7344	0.8092	0.8946					2.24	1.70	31,144	
	P	7360	0.8044	0.8930					2.28	1.73	30,254	
	C	7358	0.7973	0.8887	0.7971	0.7971	0.8023	0.7171	2.28	1.74	28,937	
	V	7377	0.8073	-	-	-	-	-	2.22	1.69	-	103

(*) *n* = the number of compounds in a set; *R*^2^ = determination coefficient; *CCC* = concordance correlation coefficient [9]; *Q*^2^*_F_*_1_, *Q*^2^*_F_*_2_, and *Q*^2^*_F_*_3_ = improved cross-validation criteria [10]; *<R_m_*^2^*>* = average rm2 metrics [11]; *RMSE* = root mean squared error; *MAE* = mean absolute error; ***F*** = Fischer F ratio; *Nac* = the number of parameters for the Monte Carlo optimization (the number of active SMILES attributes).

**Table 2:** The numbers of compounds included in the process of checking the predictive potential of the *i*-th model for the *k*-th split.

	S1	S2	S3	S4	S5	S6	S7	S8	S9	S10
**M1**	7431	1896	1809	1861	1891	1797	1846	1938	1642	1823
**M2**	1896	7499	1853	1926	1952	1872	1940	1916	1822	1931
**M3**	1809	1853	7337	1891	1790	1863	1846	1837	1817	1824
**M4**	1861	1926	1891	7404	1877	1797	1886	1873	1775	1872
**M5**	1891	1952	1790	1877	7326	1801	1805	1853	1857	1842
**M6**	1797	1872	1863	1797	1801	7332	1885	1835	1844	1826
**M7**	1846	1940	1846	1886	1805	1885	7384	1907	1855	1848
**M8**	1938	1916	1837	1873	1853	1835	1907	7558	1835	1919
**M9**	1842	1822	1817	1775	1857	1844	1855	1835	7268	1808
**M10**	1823	1931	1824	1872	1842	1826	1848	1919	1808	7377

**Table 3:** Determination coefficients were observed when testing the predictive potential of the *i*-th model for the *k*-th split and the average values and dispersion of the determination coefficients.

	S1	S2	S3	S4	S5	S6	S7	S8	S9	S10	$\bar{x}\pm \Delta x$
**M1**	0.8046	0.8191	0.8045	0.7898	0.7918	0.8105	0.8072	0.7958	0.7984	0.7873	0.8009 ± 0.0095
**M2**	0.8280	0.8239	0.8132	0.8040	0.8221	0.8283	0.8147	0.8265	0.8222	0.8271	0.8210 ± 0.0075
**M3**	0.8098	0.8048	0.8157	0.7895	0.8161	0.8194	0.8175	0.8107	0.8233	0.8206	0.8127 ± 0.0094
**M4**	0.7970	0.7901	0.7844	0.8002	0.8020	0.8065	0.8035	0.7924	0.8149	0.8146	0.8050 ± 0.0095
**M5**	0.8093	0.8226	0.8278	0.8099	0.8239	0.8233	0.8353	0.8180	0.8116	0.8406	0.8222 ± 0.0100
**M6**	0.8196	0.8227	0.8216	0.8083	0.8197	0.8240	0.8121	0.8184	0.8400	0.8204	0.8207 ± 0.0101
**M7**	0.7986	0.7932	0.8078	0.7931	0.8154	0.7982	0.8030	0.7861	0.8147	0.8166	0.8027 ± 0.0101
**M8**	0.8125	0.8227	0.8193	0.7985	0.8179	0.8240	0.8106	0.8132	0.8268	0.8282	0.8173 ± 0.0085
**M9**	0.7881	0.7970	0.8100	0.8003	0.7933	0.8262	0.8110	0.8100	0.8067	0.8177	0.8050 ± 0.0109
**M10**	0.7789	0.8005	0.8043	0.8059	0.8241	0.8053	0.8162	0.8083	0.8202	0.8073	0.8071 ± 0.0118

**Table 4:** Mean absolute error values, which were observed when testing the predictive potential of the *i*-th model for the *k*-th split.

	S1	S2	S3	S4	S5	S6	S7	S8	S9	S10
**M1**	1.67	1.66	1.67	1.71	1.71	1.64	1.65	1.69	1.67	1.63
**M2**	1.57	1.62	1.62	1.66	1.62	1.58	1.62	1.59	1.61	1.61
**M3**	1.63	1.66	1.64	1.70	1.64	1.60	1.67	1.63	1.66	1.58
**M4**	1.67	1.72	1.73	1.71	1.72	1.71	1.68	1.71	1.71	1.66
**M5**	1.63	1.61	1.60	1.67	1.62	1.60	1.61	1.66	1.67	1.56
**M6**	1.59	1.61	1.60	1.69	1.61	1.62	1.63	1.60	1.63	1.57
**M7**	1.70	1.77	1.76	1.75	1.73	1.73	1.74	1.76	1.73	1.68
**M8**	1.60	1.60	1.59	1.68	1.65	1.58	1.63	1.62	1.61	1.64
**M9**	1.73	1.73	1.76	1.78	1.78	1.72	1.73	1.70	1.74	1.67
**M10**	1.68	1.74	1.68	1.70	1.66	1.65	1.68	1.75	1.65	1.69

**Table 5:** The DCW(3.15) calculation for cyanamide (NC#N), using the correlation weights of the model obtained with split-1. DCW(3.15) = 3.6993.

SMILES Attribute	Correlation Weight
***S_k_***	***CW* (*S_k_*) **
N...........	0.9532
C...........	−0.0412
#...........	0.2896
N...........	0.9532
***SS_k_***	***CW* (*SS_k_*) **
N...C.......	0.9275
C...#.......	0.0088
N...#.......	0.6083

# triple bond.

## Data Availability

Data are available within the article or its Supplementary Materials.

## Supplementary Materials

The following supporting information can be downloaded at: https://www.mdpi.com/article/10.3390/molecules28207231/s1. The Supplementary Materials section contains details on the model calculated with Equation (3). Tables S1–S4 contain technical details for active and passive training, calibration, and validation sets, respectively.

## References

[1] Wei Y., Cao T., Thompson J.E. (2012). The chemical evolution & physical properties of organic aerosol: A molecular structure based approach. Atmos. Environ..

[2] Kuosmanen T., Zhou X., Dai S. (2020). How much climate policy has cost for OECD countries?. World Dev..

[3] Duchowicz P.R., Aranda J.F., Bacelo D.E., Fioressi S.E. (2020). QSPR study of the Henry’s law constant for heterogeneous compounds. Chem. Eng. Res. Des..

[4] Toropov A.A., Toropova A.P., Roncaglioni A., Benfenati E. (2023). Does the accounting of the local symmetry fragments in SMILES improve the predictive potential of the QSPR-model for Henry’s law constants?. Environ. Sci. Adv..

[5] Kang X., Lv Z., Zhao Y., Chen Z. (2021). A QSPR model for estimating Henry’s law constant of H2S in ionic liquids by ELM algorithm. Chemosphere.

[6] Toropova A.P., Toropov A.A. (2021). The system of self-consistent of models: A new approach to build up and validation of predictive models of the octanol/water partition coefficient for gold nanoparticles. Int. J. Environ. Res..

[7] Toropov A.A., Toropova A.P. (2021). The system of self-consistent models for the uptake of nanoparticles in PaCa2 cancer cells. Nanotoxicology.

[8] Majumdar S., Basak S.C. (2018). Beware of naïve q2, use true q2: Some comments on QSAR model building and cross validation. Curr. Comput. Aided Drug Des..

[9] Kuei Lin L. (1989). A concordance correlation coefficient to evaluate reproducibility. Biometrics.

[10] Consonni V., Ballabio D., Todeschini R. (2009). Comments on the definition of the Q2 parameter for QSAR validation. J. Chem. Inf. Model..

[11] Roy K., Kar S. (2014). The rm2 metrics and regression through origin approach: Reliable and useful validation tools for predictive QSAR models (Commentary on ‘Is regression through origin useful in external validation of QSAR models?’). Eur. J. Pharm. Sci..

[12] Toropov A.A., Toropova A.P. (2020). QSPR/QSAR: State-of-art, weirdness, the future. Molecules.

[13] Box G.E.P. (1976). Science and statistics. J. Am. Stat. Assoc..

[14] Toropov A.A., Toropova A.P. (2019). QSAR as a random event: Criteria of predictive potential for a chance model. Struct. Chem..

[15] Rakhimbekova A., Akhmetshin T.N., Minibaeva G.I., Nugmanov R.I., Gimadiev T.R., Madzhidov T.I., Baskin I.I., Varnek A. (2021). Cross-validation strategies in QSPR modelling of chemical reactions. SAR QSAR Environ. Res..

[16] Varnek A., Kireeva N., Tetko I.V., Baskin I.I., Solov’ev V.P. (2007). Exhaustive QSPR studies of a large diverse set of ionic liquids: How accurately can we predict melting points?. J. Chem. Inf. Model..

[17] Ghaedi A. (2015). Predicting the cytotoxicity of ionic liquids using QSAR model based on SMILES optimal descriptors. J. Mol. Liq..

[18] Worachartcheewan A., Nantasenamat C., Isarankura-Na-Ayudhya C., Prachayasittikul V. (2014). QSAR study of H1N1 neuraminidase inhibitors from influenza a virus. Lett. Drug Des. Discov..

[19] Kumar P., Kumar A., Lal S., Singh D., Lotfi S., Ahmadi S. (2022). CORAL: Quantitative Structure Retention Relationship (QSRR) of flavors and fragrances compounds studied on the stationary phase methyl silicone OV-101 column in gas chromatography using correlation intensity index and consensus modelling. J. Mol. Struct..

[20] Jain S., Bhardwaj B., Amin S.A., Adhikari N., Jha T., Gayen S. (2020). Exploration of good and bad structural fingerprints for inhibition of indoleamine-2,3-dioxygenase enzyme in cancer immunotherapy using Monte Carlo optimization and Bayesian classification QSAR modeling. J. Biomol. Struct. Dyn..

[21] Begum S., Achary P.G.R. (2015). Simplified molecular input line entry system-based: QSAR modelling for MAP kinase-interacting protein kinase (MNK1). SAR QSAR Environ. Res..

[22] Ahmadi S., Aghabeygi S., Farahmandjou M., Azimi N. (2021). The predictive model for band gap prediction of metal oxide nanoparticles based on quasi-SMILES. Struct. Chem..

[23] Kumar P., Kumar A., Sindhu J., Lal S. (2023). Quasi-SMILES as a basis for the development of QSPR models to predict the CO_2_ capture capacity of deep eutectic solvents using correlation intensity index and consensus modelling. Fuel.

[24] Tajiani F., Ahmadi S., Lotfi S., Kumar P., Almasirad A. (2023). In-silico activity prediction and docking studies of some flavonol derivatives as anti-prostate cancer agents based on Monte Carlo optimization. BMC Chem..

[25] Weininger D. (1988). SMILES, a Chemical Language and Information System: 1: Introduction to Methodology and Encoding Rules. J. Chem. Inf. Comput. Sci..

